# Effectiveness of rifaximin and fluoroquinolones in preventing travelers’ diarrhea (TD): a systematic review and meta-analysis

**DOI:** 10.1186/2046-4053-1-39

**Published:** 2012-08-28

**Authors:** Sanjin Alajbegovic, John W Sanders, Deborah E Atherly, Mark S Riddle

**Affiliations:** 1 School of Pharmacy, University of Washington, Seattle, WA, USA; 2 Naval Medical Research Center, Silver Spring, 20910-7500, MD, USA; 3 PATH, Seattle, WA, USA

**Keywords:** Travelers’ diarrhea, Chemoprophylaxis, Systematic review, Meta-analysis, Rifaximin, Fluoroquinolone

## Abstract

**Background:**

Recent developments related to a safe and effective nonabsorbable antibiotic, rifaximin, and identification of postinfectious irritable bowel syndrome as a frequent sequela call for a need to reconsider the value of primary prevention of traveler’s diarrhea (TD) with antibiotics.

**Methods:**

Randomized, placebo-controlled, double-blind studies evaluating the effectiveness and safety of rifaximin or a fluoroquinolone chemoprophylaxis against TD were pooled using a random effects model and assessed for heterogeneity.

**Results:**

The nine studies (four rifaximin and five fluoroquinolone) included resulted in pooled relative risk estimates of 0.33 (95% CI = 0.24–0.45, *I*^2^ = 3.1%) and 0.12 (95% CI = 0.07–0.20, *I*^2^ =0.0%), respectively. Similar rates of treatment emergent adverse events were found between antibiotic and placebo groups.

**Conclusions:**

This meta-analysis supports the effectiveness of antibiotics in preventing TD. However, further studies that include prevention of secondary chronic health outcomes among travelers to different geographic regions, and a formal risk-benefit analysis for antibiotic chemoprophylaxis, are needed.

## Background

Travelers’ diarrhea (TD) is one of the most frequent health problems encountered by individuals traveling from developed to less-developed countries [1]. Although the illness rarely results in a serious health outcome, these infections of predominantly bacterial origin can cause significant morbidity and costs resulting from incapacitation while traveling and can disrupt valuable business and leisure excursions. Furthermore, evidence is accumulating that these infections can lead to long-term health consequences, including functional bowel disorders such as irritable bowel syndrome [2]. Such recognition of the acute and chronic impacts of these infections has raised the importance of primary disease prevention.

Prevention of TD is challenging because of the ubiquitous exposure to individuals through contaminated food, water and generally unhygienic conditions in much of the developing world. Travelers are frequently counseled on preventive risk behaviors, but, despite a traveler’s best attention to such recommendations, evidence is lacking that such precautions have any protective effect [3]. Although vaccines for many of the agents commonly associated with TD are under development, this is considered a long-term solution and might likely suffer from lack of utilization as has been seen with most travel-associated vaccines [4-8].

Antimicrobial prophylaxis has been considered an option to prevent infection. In 1985, issues surrounding prophylaxis were debated during a National Institutes of Health (NIH)-sponsored consensus meeting, which concluded that routine antibiotic chemoprophylaxis should not be used because of concerns about the development of antibiotic resistance, the demonstrated efficacy of empiric therapy after the development of symptoms, and the potential for unnecessary side effects [9]. Since that meeting, studies that have examined the costs versus benefits of chemoprophylaxis for the prevention of TD have recommended against prophylaxis except in high-risk groups [10,11]. Although debate continues, the standard practice and recommendation have remained unchanged for 20 years [12-14].

Two recent developments are challenging the general recommendation against use of chemoprophylaxis. First, postinfectious irritable bowel syndrome (PI-IBS) has been recognized as an important post-TD sequela, occurring in approximately 7% of those who experience an enteric infection, particularly among those with bacterial infection and a more severe clinical presentation [15,16]. Second, rifaximin, a nonabsorbable antibiotic, has been developed and may provide a safer alternative for prophylaxis than fluoroquinolones, which are known to be quite effective but may have an unacceptable safety profile. The high volume of international travel, and, consequently, the high number of people at risk from acquiring TD, PI-IBS and other postinfectious chronic health conditions, create a potentially large burden of illness that could be prevented with the use of safe and effective chemoprophylaxis. A number of traditional and systematic qualitative reviews on the use of chemoprophylaxis, and in particular rifaximin, have been reported in the past few years and have generally favored the consideration of rifaximin for prevention of TD among high-risk groups or the risk-averse traveler [17-21]. However, those studies rarely assess the quality of the studies being reviewed and often do not result in a summary effect estimate of an intervention, which can be of further use for analytic decision-making and cost-benefit analysis. In addition, due to the nature of those reviews, they can be subject to bias and may not adequately consider disparate findings. Therefore, the objective of this study was to conduct a systematic review of the literature on the effectiveness and safety of rifaximin and fluoroquinolone antibiotics in the prevention of TD.

## Methods

This systematic review and meta-analysis was conducted according to the guidelines set forth in the *Cochrane Handbook for Systematic Reviews of Interventions*[22].

### Literature search and study selection

**Figure 1:**
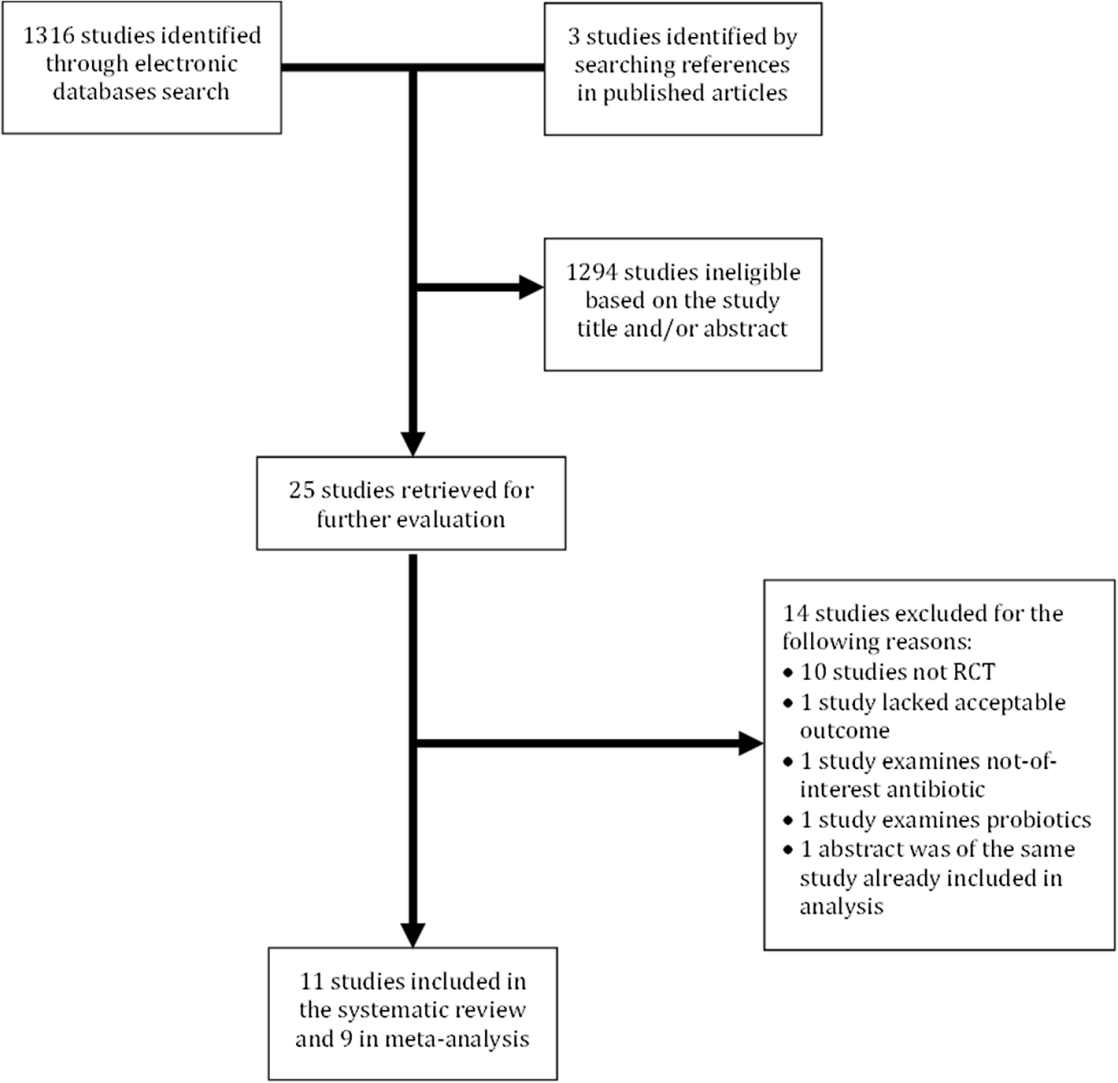
**Flow diagram of literature review, eligibility determination and inclusion in the systematic review and meta-analysis.** RCT, randomized controlled trial.

A comprehensive literature search was performed to identify all randomized, double-blind, placebo-controlled trials that evaluated effectiveness of rifaximin or a fluoroquinolone in preventing TD. Studies that examined other antibiotics were excluded because currently the only clinically relevant antibiotics for preventing TD are rifaximin and fluoroquinolones. The population of interest comprised adult civilian or military travelers. Studies were identified using the electronic databases PubMed (through April 2012) and Embase (through April 2012) and consultation with experts in the field. The following search strategy was used on PubMed: “travel* diarrh*” and “travel” (MeSH). The search was limited to studies on human subjects and published in English. Citations from retrieved articles were examined for primary outcome data completeness, and studies that reported rates of TD in treatment and control groups were included in the meta-analysis. In addition, consultation with experts in the field was used to identify any missing articles. Eligibility of all articles for systematic review and meta-analysis was confirmed by two investigators (SA and MR). The details of the literature search and study selection are presented in Figure1.

### Data abstraction and risk of bias assessment

Data were collected from each study and recorded on a data abstraction form, which was designed *a priori* and pilot-tested on three studies. Primary data abstraction was performed by an investigator (SA) and validated independently by another investigator (JS) before the data were used for statistical analysis. The primary end point of interest was the overall risk reduction in incidence of TD in treatment versus control group with the intention-to-treat (ITT) sample sets of each study. Secondary end points of interest included per-protocol (PP) risk reduction, efficacy against moderate to severe TD and safety of prophylactic antibiotics as measured by rates of drug-attributed adverse events (AEs) in treatment and control groups and/or other noted clinically significant AEs. Other information that was abstracted from each study included first author, publication name and year published, article title, study design, country of traveler/travel, year of study, proportion male, mean/median age, antibiotic dose and frequency, duration of therapy, travel purpose, and follow-up posttherapy, time of therapy initiation relative to arrival, list of exclusion criteria, definition of TD used and any other definitions of TD used (mild, moderate or severe), initial number of subjects randomized to each study arm, number of subjects that completed follow-up schedule in each study arm, overall incidence of TD at week 1 and week 2 after initiating intervention (PP and ITT data), and the incidence of moderate/severe TD (PP and ITT data).

The risk of bias assessment was performed independently by two investigators (SA and JS) using guidelines set forth in the *Cochrane Handbook*[22]. Seven domains were evaluated for bias (random sequence generation, allocation concealment, blinding of participants and personnel, blinding of outcome assessment, incomplete outcome data, selective reporting and other bias). Criteria for the “other bias” domain were discussed and specified *a priori* and included an assessment of the bias associated with exposure risk (single vs. multiple travel locations, seasonal variation of travel and the risk level of the participant) and assessment of adherence to randomized regimen. Each domain was assigned one of the following qualitative scores: “low risk,” “high risk” or “unclear risk.” In general, if there was evidence of bias, the study was deemed to be “high risk” for bias for that specific domain. An “other bias” domain of risk assessment took into consideration adherence assessment/reporting and bias associated with exposure risk. Discordant assessments for each domain of each article were discussed and adjudicated by reaching consensus among three investigators (SA, MR and JS).

### Data analysis

The proportion of subjects experiencing TD in treatment and control groups (dichotomous outcome) was used to determine the relative risk of developing TD when a chemoprophylactic antibiotic was administered versus placebo. Relative risks were then pooled independently for fluoroquinolones and rifaximin to determine the overall effect of chemoprophylactic antibiotic treatment with a 95% confidence interval (CI). Given the heterogeneity of study designs, population, and dosing regimens, a conservative approach was employed for all analyses based on a random effects model of the DerSimonian and Laird (D&L) method to reflect both within- and between-study variability. Relative risk of 1 indicated that chemoprophylaxis did not reduce the incidence of TD any more than placebo. Risk differences were similarly pooled for purposes of estimating numbers needed to treat to prevent an episode of TD while traveling.

Sensitivity analyses were performed graphically by observation of differences in pooled relative risks based on within-drug-class study characteristics and for PP versus ITT data. Incidence of moderate/severe TD was only pooled for fluoroquinolone studies since only one rifaximin study reported rates of severe TD in treatment and placebo groups. Heterogeneity was explored visually and statistically using the heterogeneity χ^2^ and the inconsistency index statistic (*I*^2^). Publication bias was assessed using the method described by Harbord *et al*. [23]. All analyses were done using Stata version 12.1 software (StataCorp, College Station, TX, USA).

## Results

### Study selection and study characteristics

Electronic database, manual reference search and consultation with experts in the field yielded 11 studies that were included in the systematic literature review and 9 that were included in a meta-analysis [24-34] (Table 1). The study by Parry *et al*. was not included in the meta-analysis because it did not define TD [29], and the study by Taylor *et al*. was a human experimental challenge study and thus was not conducted in a field efficacy setting [32]. Nine studies included in the meta-analysis had a total of 1,310 subjects, with 604 subjects in rifaximin studies and 706 subjects in fluoroquinolone studies. Four studies examined the effectiveness of rifaximin in preventing TD, and one of those studies (DuPont *et al*.) had three treatment arms (rifaximin dosed once, twice or three times daily) and one control arm (placebo dosed three times daily) [25-28]. The remaining five studies utilized a fluoroquinolone (three norfloxacin and two ciprofloxacin) and included single dose (*n* = 4) and one twice daily regimen. Overall, among nine studies, a median attack rate of 34.5% (interquartile range [IQR] = 20.3% to 53.7%) was found in the placebo groups, and attack rates were similar between the rifaximin studies (median 33.1%, IQR = 17.9% to 50.4%) and the fluoroquinolone studies (median 34.5%, IQR = 25.6% to 61.0%) (*P* = 0.32, Kruskal–Wallis test). There was greater diversity in traveler populations and geographic destinations for studies evaluating fluoroquinolone efficacy compared to those evaluating rifaximin. Duration of intervention was on average 14 days, and TD definitions were generally similar across all studies, except one (Heck *et al*. [33]), where TD was defined as ≥3 loose stools/8-hour period plus ≥1 associated symptom (vs. standard definition of TD, ≥3 loose stools/24-hour period plus ≥1 associated symptom) which may have underestimated the incidence of TD in the treatment and placebo groups.

**Table 1:** Characteristics of studies included in systematic review and meta-analysis for protective efficacy against travelers’ diarrhea

**Author**	**Country of traveler**	**Country of travel**	**Population**	**Study design**	**Antibiotic evaluated**	**Dose and frequency**	**No. of patientsin allregimens**	**Duration of therapy**	**Protective Efficacy**
Studies reviewed and included in meta-analysis									
Johnson *et al*. (1986) [24]	USA	Mexico	Language school	R, DB, PC	Norfloxacin	400 mg daily	115	14 days	88%*
Wistrom *et al*. (1987) [31]	Sweden	Africa, Asia, South America	NS	R, DB, PC	Norfloxacin	200 mg bid	115	5 to 21 days	84%*
Scott *et al*. (1990) [34]	USA/ Italy	Egypt	Military	R, DB, PC	Norfloxacin	400 mg daily	222	7 days	93%*
Rademaker *et al*. (1989) [30]	The Netherlands	Tunisia	Leisure	R, DB, PC	Ciprofloxacin	500 mg daily	53	8 days	94%*
Heck *et al*. (1994) [33]	USA	Central and South America	Volunteering	R, DB, PC	Ciprofloxacin	500 mg daily	278	5 to14 days	85%*
DuPont *et al*. (2005) [26]	USA	Mexico	Language school	R, DB, PC	Rifaximin	200 mg daily, bid, tid	210	14 days	73%*^,a^
Armstrong *et al*. (2010) [25]	USA	Turkey	Military	R, DB, PC	Rifaximin	1,100 mg daily	95	14 days	67%
Martinez-Sandoval *et al*. (2010) [28]	USA	Mexico	Language school	R, DB, PC	Rifaximin	600 mg daily	201	14 days	68%*
Flores *et al*. (2011) [27]	USA	Mexico	Language school	R, DB, PC	Rifaximin	550 mg daily	98	14 days	28%
Other studies of relevance reviewed but not included in meta-analysis									
Parry *et al*. (1994) [29]	UK	Nepal	Himalayan expedition	R, DB, PC	Ciprofloxacin	250 mg daily	21	28 days	NA
Taylor *et al*. (2006) [32]	NA	NA	Volunteers	R, DB, PC	Rifaximin	200 mg tid	25	3 days	NA

^a^Rifaximin groups combined. Note: Studies are listed in chronological order by an antibiotic studied. bid = twice daily; tid = three times daily; R, DB, PC = randomized, double-blind, placebo-controlled; NA = not available; NS = not specified. *Statistically significant.

### Risk of bias assessment

**Figure 2:**
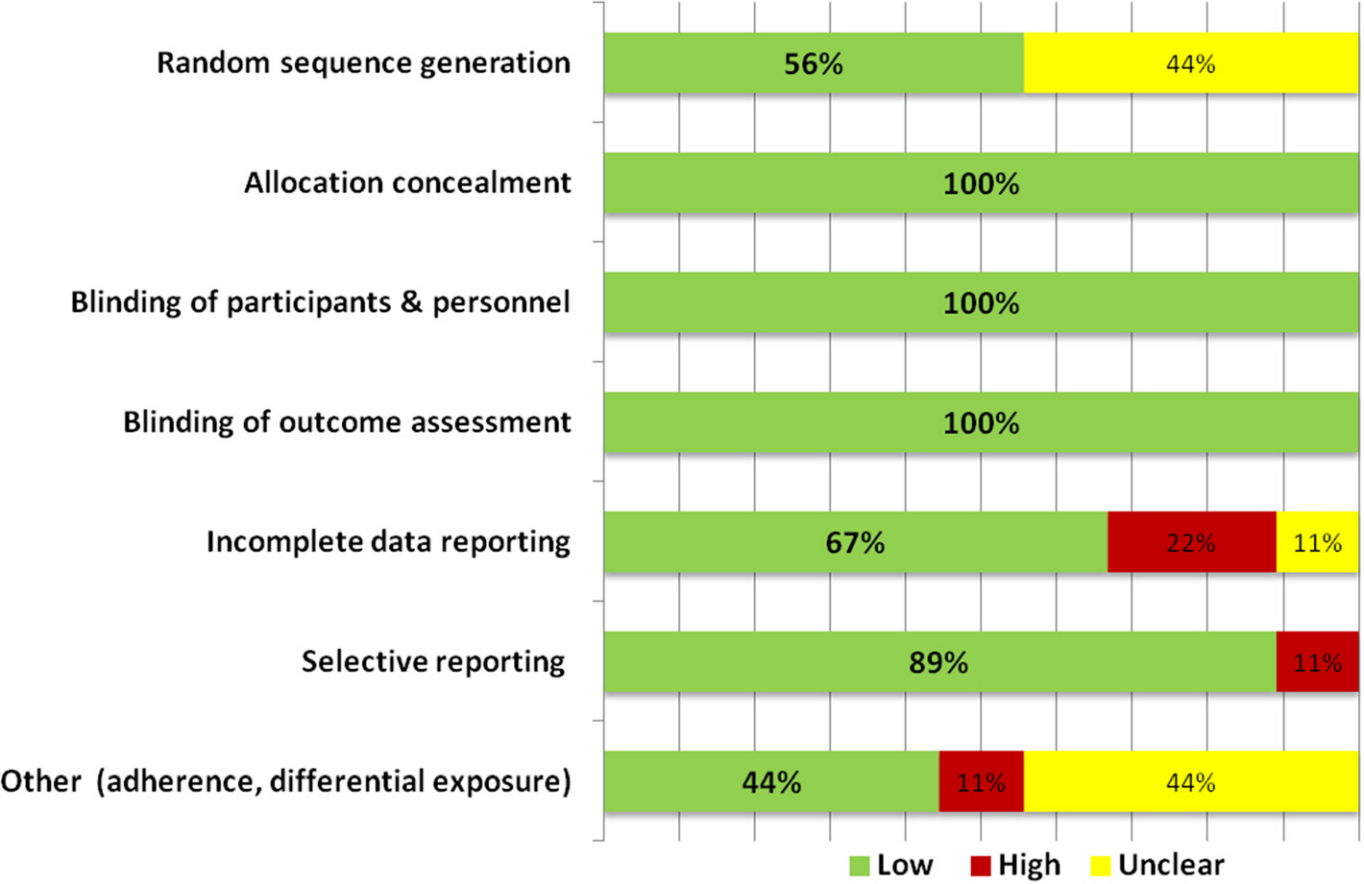
**Frequency distribution of assessment of bias scoring among included studies in meta-analysis ( *n* = 9).**

All studies were deemed to have “low risk” of bias in domains of allocation concealment, blinding of participants and personnel, and blinding of outcome assessment (Figure2). Although no study specified whether blinding of outcome assessment took place, investigators deemed that, owing to the relatively objective nature of the clinical outcome and standard use of definitions, low risk for bias existed even if the outcome assessment was not blinded. Four studies did not specify the process of randomization and were therefore assigned “unclear risk” for random sequence generation domain. DuPont *et al*. [26] had significantly different attrition rates between treatment and control groups, and Heck *et al*. [33] reported a relatively high attrition rate that was not adequately handled/reported; therefore, both studies were assigned “high risk” for the incomplete outcome data domain. Unclear risk in incomplete outcome data domain was assigned to the study by Armstrong *et al*. because of the low number of stool specimens collected, an end point that was prespecified in the Methods section of the study [25]. A single study had high risk for bias in selective data reporting domain due to the fact that an ITT outcome for the primary end point was not available [33].

### Primary outcome: prevention of TD in ITT analyses

**Figure 3:**
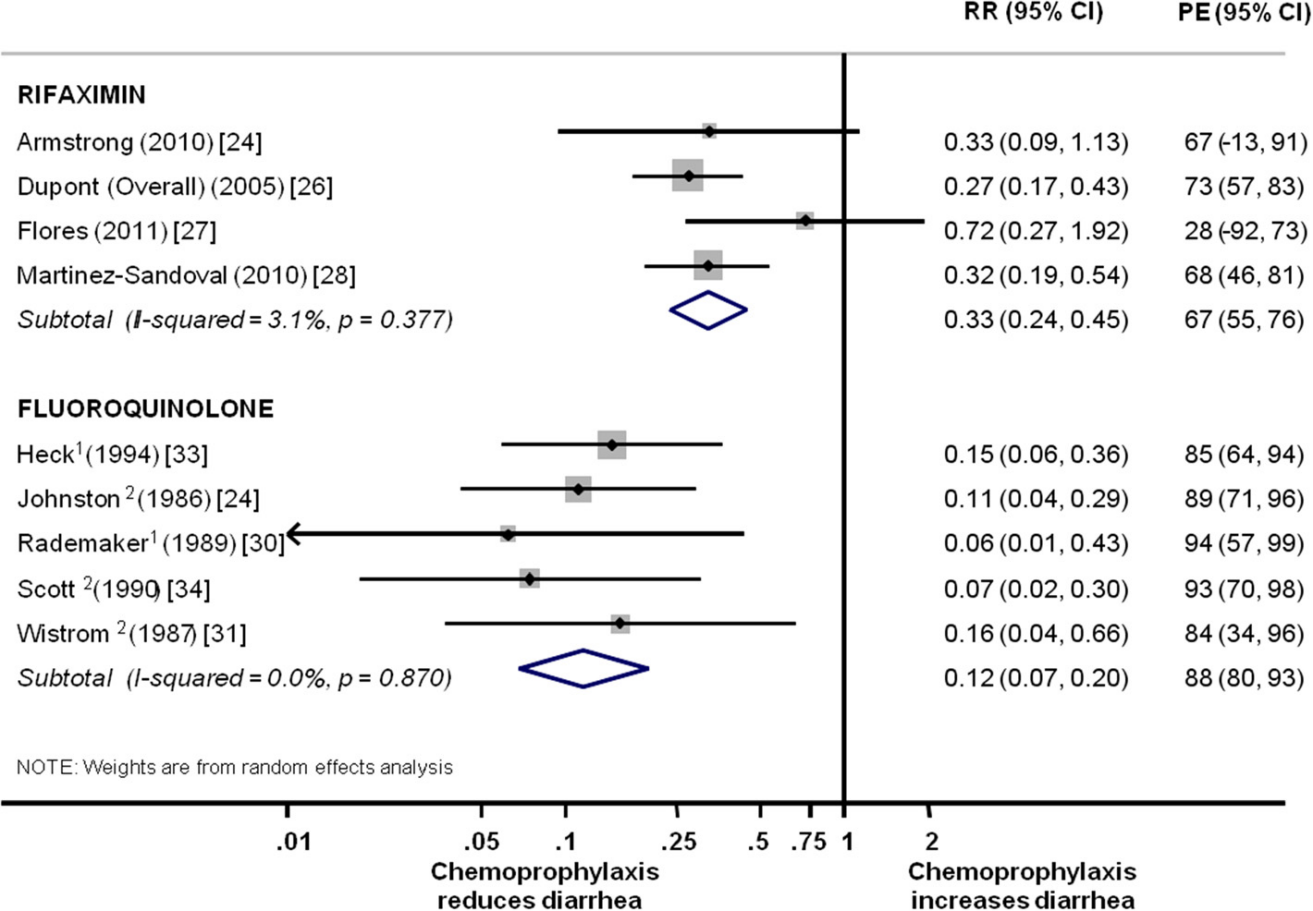
**Pooling of studies by drug class for intention to treat analysis.** RR = relative risk; PE = protective efficacy; CI = confidence interval. 1. Regimen using ciprofloxacin. 2. Regimen using norfloxacin.

ITT data from studies examining fluoroquinolones and rifaximin were pooled by drug class to determine whether antibiotic chemoprophylaxis was effective in preventing TD (Figure3). Among the rifaximin studies, two of the four studies showed a statistically significant treatment effect and one demonstrated marginal statistical significance. There was no observed significant difference of risk reduction among dosing regimens in the DuPont *et al*. study, and the overall pooled D&L effect (relative risk [RR]) estimate for all studies combined was 0.33 (95% CI = 0.24 to 0.45), equating to a protective efficacy of 67% (95% CI = 55% to 76%) favoring chemoprophylaxis (heterogeneity χ^2^ = 3.09, *P* = 0.377; *I*^2^ =3.1%). In terms of absolute risk reduction, pooled D&L summary estimates found that rifaximin chemoprophylaxis decreased TD attack rates by a mean of 22.1% (95% CI = 6.3% to 37.9%) equating to a number needed to treat (NNT) of 4.5 people (95% CI = 2.6 to 15.9) who needed chemoprophylaxis to prevent one episode of TD. All five of the studies evaluating fluoroquinolone-based regimens showed a statistically significant effect with a pooled D&L effect RR estimate of 0.12 (95% CI = 0.07 to 0.20), equating to a protective efficacy of 88% (95% CI = 80% to 93%) favoring chemoprophylaxis (heterogeneity χ2 = 1.25, *P* = 0.87; *I*^2^ =0.0%). Risk reduction for fluoroquinolone-class antibiotics was estimated with a D&L pooled mean of 35.5% (95% CI = 21.2% to 49.8%), equating to a NNT of 2.8 (95% CI = 2.0 to 4.7). For both drug classes, there was no indication of publication bias (rifaximin, *P* = 0.63; fluoroquinolones, *P* = 0.56).

Further exploration of the studies by drug class was conducted and based on a subanalysis of the effect of pooling data from stratification of the Dupont *et al*. study including once-daily treatment arm, treating three different treatment arms as if they were independent studies, and combining results from three different treatment arms [26]. When slightly different pooled relative risks were examined, all analytical strategies still favored chemoprophylaxis with rifaximin over placebo (daily: RR = 0.34, 95% CI = 0.22 to 0.51; independent: RR = 0.32; 95% CI = 0.24 to 0.43; combined: RR = 0.33; 95% CI = 0.24 to 0.45). On the basis of the similarity of these effect estimates, a decision was made to use a combined estimate from all three dosing regimens for purposes of further analysis. Further exploration of heterogeneity within each drug class was challenging, given the small number of studies. The study by Flores *et al*. stands out compared to the other studies included in this systematic review and differed only in nonsummer enrollment period compared to the other studies conducted in similar populations in Mexico [27].

### Secondary outcomes: prevention of TD in PP analyses and moderate to severe disease

Analysis of efficacy in the PP sample set was available for all four studies in the rifaximin drug class. (Note: PP data were provided by one of the coauthors, MR, for the Armstrong *et al*. study [25].) The D&L summary pooled RR effect estimate was 0.27 (95% CI = 0.17 to 0.43) and tended toward moderate heterogeneity (heterogeneity χ^2^ = 5.43, p = 0.14; I^2^ = 44.7%). Only three studies within the fluoroquinolone drug class included outcome data from subanalysis of moderate to severe TD and compared to placebo, though definitions varied across these studies. Rademaker *et al*. defined severe diarrheal illness as “symptoms of vomiting and/or fever >38°C,” whereas Heck *et al*. and Johnson *et al*. defined confirmation of moderate to severe TD by the presence of *Shigella* spp. [24,30,33] and outcome events of moderate to severe TD was low (five, two and three cases, respectively, for both treatment and placebo groups combined). Considering these limitations, however, a pooled D&L relative risk against moderate to severe TD of 0.51 (95% CI = 0.095 to 2.71) with low heterogeneity (heterogeneity χ^2^ = 2.08, *P* = 0.354, *I*^2^ = 3.8%) was found and represented poorer efficacy compared to prevention against all levels of TD severity.

### Secondary outcome: safety and adverse events

The incidence of treatment emergent AEs was not reported consistently across studies. AEs of note occurred in subjects taking fluoroquinolones. Treatment emergent AEs reported in studies examining fluoroquinolones included a rash that resolved after norfloxacin was discontinued [24], enlargement of parotid glands [31], and sunburn in a subject taking ciprofloxacin [30]. Otherwise, all reported rates of AEs were similar between treatment and control groups, and no clinically significant events developed during chemoprophylaxis with rifaximin or a fluoroquinolone.

### Review of studies not included in meta-analysis

Two clinical trials not included in the meta-analysis should be considered in review of the potential effectiveness of antibiotic chemoprophylaxis for the complementary evidence they may bring forth. In a letter to the editor, Parry *et al*. described a small, double-blind, randomized, placebo-controlled trial of 250 mg of ciprofloxacin compared to placebo in a group of British mountaineers trekking in the Himalayas [29]. The primary end point was not a case definition of TD, but rather a decision by the expedition medical officer to break the code and to stop the trial medication. Among 21 participants (10 ciprofloxacin and 11 placebo) who completed the study, 6 of 11 placebo recipients met the end point criteria, whereas none of the 10 taking ciprofloxacin met the end point (*P* < 0.05). It was also noted that gastrointestinal complaints such as loose stools, cramping and nausea were decreased among those taking ciprofloxacin compared to placebo.

Taylor *et al*. reported the efficacy in a human experimental challenge study in which 15 volunteers were treated with 200 mg tid rifaximin and 10 were randomized to placebo in a *Shigella flexneri* 2a (2457 T) challenge at 1,500 colony-forming units. In this study, there were no cases of shigellosis in the rifaximin group, compared with 6 of 10 in the placebo group becoming ill (*P* = 0.001) [32].

## Discussion

This systematic review summarizes the findings of several studies showing a comparative advantage of antibiotic chemoprophylaxis for the prevention of TD. With respect to rifaximin chemoprophylaxis, two studies (Flores *et al*. and Armstrong *et al*.) did not show that chemoprophylaxis with rifaximin reached a statistically significant difference in preventing TD compared to placebo [25,27]. In both studies, the incidence of TD in the control group was relatively low (8 of 48 (17%) and 9 of 47 (19%), respectively), which could have explained the findings given that the sample size calculations were based on the expected incidence of TD of 40% and thus may have been too small to detect the true effect of rifaximin in preventing TD. Furthermore, though studies utilizing rifaximin were limited to only two regions, there appeared to be a consistent effect of protection at 67% (D&L 95% CI = 55% to 76%) with little heterogeneity (heterogeneity χ^2^ = 3.09, *P* = 0.377; *I*^2^ =3.1%), and the study by Taylor *et al*. suggests that rifaximin may be effective against more invasive pathogens occurring in other common travel destinations [32]. However, until such studies are done in field settings (and against a broader range of invasive pathogens to include nontyphoid *Salmonella* and *Campylobacter*), routine chemoprophylaxis against TD with rifaximin may not be appropriate if a traveler is going to destinations where diarrheagenic *Escherichia coli* are less common.

The effectiveness of fluoroquinolone antibiotics would appear to be greater than rifaximin, which is not surprising, given the broader spectrum of coverage and systemic distribution of this drug class. Pooled estimates of fluoroquinolone efficacy were 88% (95% CI 80% to 93%) with little evidence of heterogeneity (heterogeneity χ^2^ = 1.25, *P* = 0.87; *I*^2^ = 0.0%), though less effective when moderate to severe TD as an outcome is considered (summary D&L efficacy of 49%). It is important to note that studies examining fluoroquinolones were not as current as the studies that examined rifaximin (publication 1986 to 1994 vs. 2005 to 2011). As sanitation and hygiene conditions improve, resistance to fluoroquinolones emerges, and geographical patterns of resistance change (and keep evolving), older data citing fluoroquinolone use for chemoprophylaxis may become obsolete. The data from older studies of the use of fluoroquinolones may be less contemporaneous, but it is still valuable as it adds perspective and places newer data from rifaximin studies into context.

Although such a meta-analysis of the efficacy of antibiotics for TD prevention is interesting, the results do not necessarily compel one to embrace chemoprophylaxis as a potential solution. Even in the face of efficacy data for safer antibiotics such as rifaximin, the prevailing consensus is against widespread use exemplified by Dr. Gorbach, Chairman of the 1985 NIH Consensus Development Panel, who wrote an accompanying editorial to the first report of rifaximin diarrhea prophylactic efficacy in 2005 [13]. Gorbach’s concerns included potential unintended adverse consequences, such as safety issues, uncertain protection against invasive pathogens, and microbiologic adverse effects, that may not yet be apparent in the small number of studied individuals. Dr. Gorbach concluded with the statement, “Rapid and judicious treatment of diarrhea, not antibiotic prophylaxis, is the best recommendation for most travelers.”

Although these concerns still prevail today, a new consideration regarding the potential risk of postinfectious chronic health consequences of TD has arisen. Serious sequelae such as reactive arthritis [35] and Guillain-Barré syndrome [36] have long been known to be associated with infections causing TD, but they are relatively infrequent. Most notable has been the accumulating evidence associating TD with postinfectious irritable bowel syndrome. Two separate systematic reviews have now been published which conclude that roughly 1 of 11 people who develop acute diarrheal infection may go on to develop PI-IBS [15,16]. Other studies are also reporting TD risk with other common postinfectious functional gastrointestinal disorders [37-40]. With TD specifically, there have been six studies among traveler populations, all of which have shown an increased risk of PI-IBS among travelers who develop TD compared to those who do not develop TD [39-44]. Factors which appear to be associated with increased risk of PI-IBS include fever, illness severity, duration, infection with an invasive pathogen, and concomitant acute stress. Furthermore, this risk remains elevated for at least 3 years after the infection [16] and has been described to persist in 57% after 6 years in one study and 76% after 5 years in another [45,46].

Thus, the recognition of both the acute and chronic consequences associated with TD diarrhea may change the risk-balance and value equation for antibiotic chemoprophylaxis. A recent economic analysis by Lundkvist *et al*., who evaluated the potential cost-effectiveness of a vaccine against enterotoxigenic *E. coli*, described the cost of a TD event of $1,460 or $1,996 for a leisure or business traveler, respectively (including value of travel, value of time, and medical costs) [47]. With regard to IBS, in a review of 18 economic studies conducted in the United States and United Kingdom, direct and indirect cost per patient-year were estimated between $700 and $12,000 (2002) [48]. In this systematic review, we found a NNT with chemoprophylaxis to prevent TD during travel of 2.8 (95% CI = 2.0 to 4.7) for fluoroquinolones and 4.5 (95% CI = 2.6 to 15.9) for rifaximin. A back-of-the-envelope calculation would suggest that to prevent the cost of acute disease, a single-dose, 14-day regimen of rifaximin 550 mg (at $22.61 per dose, $316.58 regimen) would provide a net benefit of $35 for a leisure traveler ($1,460 – (4.5 × $316.58)) and a $571 net benefit for the average business traveler to an average-risk region. Fluoroquinolones at less than $1.00 per dose (ciprofloxacin) would appear to be even more cost-effective using a simplistic calculation. However, such cost savings need to be balanced by the cost of potential AEs associated with antibiotics. Interestingly, in this systematic review, there were no treatment emergent-related AEs reported for any of the studies, though the inclusion of risk of *Clostridium difficile* infection and fluoroquinolone-related tendonopathies ought to be considered more formally in an economic analysis. If one were to more comprehensively consider the value added in preventing PI-IBS and other chronic health consequences, the benefits of chemoprophylaxis in acute and chronic disease prevention may appear to outweigh the risks associated with chemoprophylaxis. Clearly, future studies are needed to better define the economic cost associated with PI-IBS and whether chemoprophylaxis with rifaximin or fluoroquinolones can be used safely to prevent such sequelae.

The present review includes a comprehensive literature search, *a priori* inclusion and exclusion criteria, standardized data abstraction, risk of bias scoring, and current analytic methods, all of which reduce the potential bias in the resultant population of studies used for analysis. However, the small number of studies and the lack of studies conducted among a variety of study population types and geographic regions limit the broad application of these results beyond young healthy travelers and, for rifaximin, to regions outside Central America, where diarrheagenic *E. coli* may not predominate. Though comprehensive, our search strategy might not have identified all eligible studies. We did not find heterogeneity in study effect estimates, but the studies evaluating fluoroquinolones were not current and thus may have been subject to changes in traveler population demographics and secular trends of antimicrobial resistance. Therefore, translation of results from this type of controlled setting to other populations who may be less healthy or subject to different travel or treatment environments need to be validated by additional studies. This systematic review afforded an objective assessment of study design and risk of bias for the population of chemoprophylaxis trials included. Although the risk of bias for most domains was considered low, the authors felt that there was incomplete data reporting for some studies, which could have included important secondary outcomes related to per-protocol or efficacy evaluable analyses and efficacy against moderate to severe disease. In addition, it was noted that not all studies included information on how they ensured or measured treatment adherence and what potential effect such nonadherence may have had on study results. Given the known problem of medication adherence with malaria chemoprophylaxis, such an assessment for TD adherence within these trial settings would be informative [49,50]. Last, some studies had significant lost to follow-up rates, which, though generally balanced across treatment arms, raises further concern about self-selection bias and the generalizability of these results. Future studies ought to consider better methods to ensure avoidance of dropouts and losses to follow-up, which can be a challenge in a travel setting.

## Conclusions

It is crucial that clinicians, including pharmacists who practice in a travel clinic setting, stay well-informed on geographical patterns, itineraries, traveler behaviors and areas where the risk of developing TD is high (high-risk areas). If a traveler is planning on taking a short-term trip (≤14 days in duration) to an area where the risk for developing TD is high and diarrheagenic *E. coli* pathogens predominate, the data reviewed in this study support the authors’ opinion that routine chemoprophylaxis with rifaximin may be a reasonable choice if the patient does not have any contraindications for use.

## Competing interests

MR has served on scientific advisory boards for and received research grants from Salix Pharmaceuticals (Morrisville, NC, USA). All other authors declare that they have no competing interests. This study was conducted by each author without internal or external funding.

## Authors’ contributions

SA and MR participated in design of the study, conducted literature search, and contributed to manuscript development. SA conducted statistical analysis and abstraction of data. JS participated in quality review, validation of abstracted data, and significant manuscript edits. DA participated in design and coordination of study and helped draft the manuscript. All authors read and approved the final manuscript.

## Disclaimer

The views expressed in this article are those of the author and do not necessarily reflect the official policy or position of the Department of the Navy, Department of Defense or the U.S. Government. This is a U.S. Government work. There are no restrictions on its use.

## Copyright statement

Two of the authors (JS and M.R.) are employees of the U.S. Government and military service members. This work was prepared as part of official duties. Title 17 USC § 105 provides that “Copyright protection under this title is not available for any work of the United States Government.” Title 17 USC § 101 defines a U.S. Government work as a work prepared by a military service member or employee of the U.S. Government as part of that person’s official duties.

## Human subjects protection

This is not human subjects research and therefore is exempt from Institutional Review Board review.
